# Does national intellectual capital matter for shadow economy in the Southeast Asian countries?

**DOI:** 10.1371/journal.pone.0267328

**Published:** 2022-05-11

**Authors:** Toan Pham-Khanh Tran, Phuc Van Nguyen, Quyen Le-Hoang-Thuy-To Nguyen, Ngoc Phu Tran, Duc Hong Vo

**Affiliations:** The CBER–Research Centre in Business, Economics & Resources, Ho Chi Minh City Open University, Ho Chi Minh City, Vietnam; University of Milano–Bicocca: Universita degli Studi di Milano-Bicocca, ITALY

## Abstract

Understanding the determinants of the shadow economy plays a vital role in formulating policies for economic growth and development, particularly for the Southeast Asian countries–a new economic force for a global economy. The key drivers of a shadow economy, such as institutional quality, taxation, government expenditure, are widely examined. However, the effect of national intellectual capital, which affects macroeconomic indicators, on the shadow economy has largely been ignored in the existing literature. Our paper examines this critical link and its causality relationship for eight Southeast Asian countries from 2000 to 2017. This paper uses the dynamic ordinary least squares (DOLS) and fully modified ordinary least squares (FMOLS), which allow cross-sectional dependence and slope homogeneity in panel data analysis. Empirical findings from this paper indicate that national intellectual capital impacts negatively and significantly the shadow economy size. This finding implies that enhancing national intellectual capital reduces the shadow economy size. These two forces lead to enhanced economic growth. Our Granger causality tests confirm a bi-directional relationship between national intellectual capital and the shadow economy. As a result, policies targeted to reduce the shadow economy size can now include the accumulation of national intellectual capital, particularly for Southeast Asian Countries.

## 1. Introduction

Shadow, informal, or unofficial economy exists as a pervasive economic feature across nations [1]. A shadow economy appears to be expanding, especially in countries where weak institutions cannot strengthen the proper functioning of the market mechanism [2, 3]. Measuring the size of the shadow economy is a challenging task for economists [4]. However, albeit imperfect [5], estimate the shadow economy size for various countries worldwide. In addition [6], has recently developed the index of national intellectual capital (INIC) for many countries globally. This pioneering index is generally considered advanced and suitable for international comparison. Interestingly, the INIC covers many macroeconomic indicators publicly available for most countries worldwide.

Previous studies have found many determinants of the shadow economy, including unemployment [7, 8]; tax burden [9, 10]; corruption [11–13]; trade openness or globalization [14]. However, our literature review indicates that the relationship between national intellectual capital and shadow economy has largely been neglected in existing empirical studies. A potential explanation for this lack of empirical evidence is that national intellectual capital is a fuzzy concept [15]. More importantly, measuring national intellectual capital is limited and incomparable across countries because of data limitations and significant judgement [6]. Nevertheless, the established literature has considered that national intellectual capital affects economic growth [15, 16], which affects shadow economy size [17, 18]. Notably, the important strand of the digital economy, known as “Industry 4.0” [19], considers that national intellectual capital is an essential feature to enhance the competitive advantage of a nation [16, 20]. This digital economy contributes to wealth creation [15, 21] and impacts economic activities, including the shadow economy. In addition, the relationship between national intellectual capital and the size of the informal economy can be explained through the components of national intellectual capital, including human capital, structural capital, and relational capital.

Countries in the Association of Southeast Asian Nations (ASEAN) provide a fruitful context to examine this critical link and its causality relationship. The size of the shadow economy in these ASEAN countries is approximately equal to 30.24 per cent of the national GDP. The smallest shadow economy of 13.1 per cent of the GDP of the region belongs to Singapore, whereas the largest size of 41.9 per cent of GDP was in Thailand in 2000 and 2017 [5]. Meanwhile, the ASEAN countries have been witnessing impressive economic growth for the last 20 years, and these countries are now fundamental forces for a global economic recovery after the current Covid-19 pandemic. However, these countries’ economic indicators on intellectual capital, education, and the knowledge-based economy are well below expectations. National intellectual capital appears to be an essential element for sustainable economic growth and development. [22] has considered that investing in national intellectual capital appears to be one of the best solutions for the ASEAN countries to enhance their competitiveness and ensure sustainable growth and development.

The main objective of this study is to examine the effects of national intellectual capital on the shadow economy across countries globally. To achieve our objective, we apply the index of national intellectual capital (INIC) developed by [6] to measure the level of national intellectual capital. A sample of 8 ASEAN countries is used during the 2000–2017 period. This study uses the dynamic ordinary least squares (DOLS) and fully modified ordinary least squares (FMOLS) techniques to investigate the potential link between national intellectual capital and the shadow economy. Findings from this study confirm that enhancing national intellectual capital is associated with reducing the shadow economy size. In addition, results from the Granger causality tests also confirm a bi-directional relationship between national intellectual capital and the shadow economy.

Given the importance of national intellectual capital and shadow economy in Southeast Asian countries, this study contributes to the existing literature on the following grounds. *First*, to the best of our knowledge, this is the first paper to investigate the link between national intellectual capital and shadow economy and their causality relationship in the ASEAN countries. *Second*, we focus on a long-term relationship between national intellectual capital and the shadow economy using the dynamic least square (DOLS) and fully modified least square (FMOLS) methods. Furthermore, pooled mean group (PMG) method is also used for robustness check. *Third*, we focus exclusively on the Southeast Asian nations from 2000 to 2017 to provide direct policy implications for the governments.

The study is structured as follows. Following this introduction, section 2 presents and synthesizes the literature review. Section 3 discusses data and research methods. The results are presented and discussed in section 4, followed by policy implications in section 5 of the paper.

## 2. Literature review

### 2.1 Shadow economy, measurements, and determinants

The term “shadow economy” is often named as informal sector or economy [23, 24] the underground economy [25, 26], the undeclared economy [27, 28]. There is an adequate, coherent and commonly accepted definition of the shadow economy. It is crucial to understand the content of a specific term.

Most scholars agree that the shadow economy is inevitable because various economic activities cannot be counted in official accounts [29, 30]. In line with this perspective [31], consider that the shadow economy includes all unregistered economic activities that would have added to the national GDP if these activities had been reported. Recently [10], introduced a narrower definition of the shadow economy, which refers to all legal, economic, and productive activities contributing to official GDP if recorded.

The shadow economy estimation techniques can be classified into three main categories: direct, indirect, and multiple indicators multiple causes (MIMIC) [32]. First, in the direct approaches, studies employ surveys, interviews, or tax auditing methods to estimate the size of the shadow economy [33–35]. Second, studies that fall into the indirect approaches based on some economic indicators to make assumptions about the shadow economy. For example [36], use five macroeconomic indicators to estimate the size of the shadow economy. These indicators include currency demand [31, 37]; discrepancies between national income and expenditure statistics [24, 38]; discrepancies between official and actual labour force [39, 40]; transactions indicator [28], and electricity consumption indicator [41, 42]. Third, the size of the shadow economy can be estimated using a set of various determinants and effects of the shadow economy. This method is called the MIMIC method [43].

Tax evasion and institutional quality are considered the leading causes of the shadow economy [44]. The theory of tax evasion identifies the economic agents who are not willing to pay high taxes if they do not receive the equivalence of high-quality public services. As a result, they would conduct their economic activities in a shadow economy to evade a high tax and social welfare system [45]. Institutional economics claim that the low institutional quality is the main reason for informal economic activities [12] due to the lower labour costs in the shadow economy compared with the official economy [43]. Besides tax evasion and institutional quality, other determinants of shadow economy have been pointed out, including tax and social contribution burden [46, 47], regulation of the labour market [48, 49], low public tax morale [50].

### 2.2 National intellectual capital and its measurements

The concept of intellectual capital was initially generated from the firm level for determining the firm’s market value [51]. However, gradually it has been extensively developed and modified on the national scale [52, 53]. This shift is driven by the idea that intellectual capital is crucial for economic growth and development and the competitiveness of nations as it is for firms [54].

The concept of national intellectual capital has been explained in previous studies. However, there is no agreed definition of national intellectual capital due to a lack of coherent theory and dependable measurement models [54]. National intellectual capital was described as all the available intangible resources to the country that can be combined to generate future benefits [55]. [15] argues that nation intellectual capital, including the latent values of individuals, businesses, institutions, communities, and regions, is an existing resource for establishing and shaping national prosperity. Futhermore [16], describe national intellectual capital as “knowledge, wisdom, capability, and expertise", determining future development. Even if definitions used by academics are different, the fundamental assumption underlying national intellectual capital is the significance of intangible resources and their impact on economic and social development [54].

Despite the widely recognized importance of national intellectual capital, its assessment and measurement are tricky because of the fuzzy concept [15, 56]. Nevertheless, measuring national intellectual capital has been explored in previous studies using various methods [15, 16, 57, 58]. National intellectual capital is often measured as an index, which is estimated by aggregating the values of its structural parts. However, these methods have many fundamental issues, including the limitation of required data, the significant use of personal judgement from participants and the lack of comparable comparison across countries [6].

Recently [6], initiated and applied a new index of national intellectual capital (INIC). This index is arguably considered simple, quantifiable, relevant to the current conditions of various economies and suitable for international comparison across nations. This new index of measuring national intellectual capital is considered an advanced index compared with other national intellectual capital measurements. Moreover, this index can be estimated and updated from publicly available data. The index can also be used to compare the level of national intellectual capital across nations and years. With these advantages, this index is used in this paper. The INIC index consists of three key components, including (1) human capital, (2) structural capital, and (3) relational capital. In their applications, the authors have used various indicators as proxies for human capital, structural capital, and relational capital. These critical data of indicators are collected from the World Development Indicators database. The principal component analysis is used to derive the INIC index, which is used as a proxy for a level of national intellectual capital.

### 2.3 The relationship between national intellectual capital and the shadow economy

The current studies on the shadow economy mainly focus on employment, taxation, and institutional quality. However, previous studies have neglected to examine other factors such as national knowledge capital, especially in the knowledge-based economy and the 4.0 technology revolution. The national intellectual capital concept can be considered the endogenous economic growth theory principles, emphasising knowledge and technology in economic growth and development. Many economists have utilized intangible inputs in their academic research, including human capital [59]; R&D investment [60]; social capital [61]; information technology development [62].

The above empirical studies have examined the impact of specific components of national intellectual capital on economic growth. However, the role of national intellectual capital in the shadow economy has not been investigated. We consider that national intellectual capital might be a key driver for the shadow economy for the following reasons. First, national intellectual capital is considered a significant contributor to sustainable economic growth development [16, 21], which in turn creates jobs, reduces poverty [53, 63]. Second, economic growth affects the shadow economic size [17]. Third, the dualist theory, rooted in [64] study, confirms that the shadow economy is the residual and the expansion in the official economy, which may reduce the size of the shadow economy [65].

Furthermore, the nexus between national intellectual capital and the shadow economy can be explained through the structural components of national intellectual capital. Therefore, human capital is one of the critical components of INIC. In the development of the INIC index, human capital is proxied by three distinct macroeconomic indicators, including (i) school enrolment (tertiary), (ii) school enrolment (secondary), and (iii) government expenditure on education. [66] use human capital as proxy of education. Their findings indicate that the improvement in education has resulted in a smaller shadow economy in the urban area of Tirana, Albania. [67] examine the impact of government spending on education on the shadow economy for 162 nations during the 1999–2006 period. Public spending on education and the percentage of educational attainment of the adult population for primary, secondary, and higher levels of education is used. The empirical results show that educational variables reduce the shadow economy size. Intelligence Quotient (IQ) score was used as educational attainment to examine the impact on the size of the shadow economy in the [68] study. The results indicate that an increase in IQ score by one standard deviation decreases the size of the shadow economy by about 8.5 percentage points of GDP. In addition, a higher educational level determines a higher level of understanding and respecting the laws. This improved understanding is associated with a smaller economic and financial crime and the shadow economy [69]. [67] argues that human capital negatively affects emerging shadow activities. People with a good level of education will understand the social norms and orders and the risks related to breaking the law. As such, they will not join economic activities in the shadow economy.

Moreover, inward foreign direct investment (FDI) is generally used as a proxy for relational capital–one component of intellectual capital. This type of capital flow might be a driver of the shadow economy. FDI inflow is an additional capital from international companies that can benefit the official economic activities [70] and improve host countries institutional quality [71, 72]. Furthermore, FDI can improve the efficiency of domestic production [73] due to the spillover effects from knowledge and technology. In this context, FDI might be a potential determinant of official activities to the detriment of the shadow economy. A negative relationship between FDI and the shadow economy has been reported in previous studies [74, 75]. Using panel data for 19 developing Asian countries from 2002 to 2015 [75], confirm that FDI inflows decrease the size of shadow economy through institutional improvement. Futhermore [76], confirm the negative impact of FDI inflows on the shadow economy. Besides, export is generally used as a proxy for relational capital. [77] argue that the inverted-U shape association between export (export diversification, export quality) and the shadow economy does exist. This finding indicates a tipping point of export diversification and export quality. Thus, export diversification and export quality reduce the shadow economy beyond the tipping point.

Our empirical review reveals that previous studies focus on a specific type of intangible resources. No comprehensive study has been conducted to incorporate all critical aspects of national intellectual capital. This research gap warrants our analysis to provide empirical evidence on the effects of national intellectual capital on the shadow economy size and their causality relationship for the eight Southeast Asian countries.

## 3. Research methodology and data sources

### 3.1 Research methodology

This study examines the impact of national intellectual capital on the size of the shadow economy in 8 ASEAN countries during the 2000–2017 period. The following general equation is used:

$$SE_{it}=\beta_{0}+\beta_{1}INIC_{it}+\beta_{4}X_{it}+\varepsilon_{it}$$ (1)

in which *i* and *t* represent a country and time, respectively. SE denotes a shadow economy size (a per cent of GDP) from [5] study. The MIMIC method is used to estimate the shadow economy size. The INIC represents a national intellectual capital calculated based on [6] approach. This newly developed index consists of three main components, including (1) human capital, (2) structural capital, and (3) relational capital.

For the control variables (Xj), the study employs economic growth (GDP per capita), trade openness (TR), bank credit (CE), government expenditure (GE), and inflation (INF). These variables are selected based on previous empirical analyses, including [43, 76].

The study begins with a correlation analysis between variables for the estimation procedure. Then, the residual cross-sectional dependence check is performed to establish whether a cointegration check is required in the analysis. The panel unit root and panel cointegration tests are examined in this study. Based on the findings on the cointegration from these tests, the dynamic OLS (DOLS) and the fully modified ordinary least squares (FMOLS) estimations are used to examine the effects of national intellectual capital on the shadow economy.

We also use the pooled mean group (PMG) estimation as the robustness analysis to ensure that our empirical results are robust. The main characteristic of PMG is that it allows short-run coefficients, including the intercepts, the speed of adjustment to the long-run equilibrium values and error variances to be heterogeneous country by country. In contrast, the long-run slope coefficients are restricted to be homogeneous across countries. Given that the countries in the sample are within the same regional economic bloc, the assumption of a homogeneous long-run estimate is plausible. However, these countries may be bonded by the same trade terms, laws, monetary policy, while the short-run estimates will differ due to country-specific economic and institutional differences.

Auto-regressive distributed lag (ARDL) model’s [78] unrestricted specification for the dependent variable SE (*y*) is expressed through Eq (2).


$$ny_{\mathrm{it}}=\sum_{j=1}^{p}\theta_{\mathrm{ij}}ny_{i,t‐j}+\sum_{j=0}^{q}\gamma_{\mathrm{ij}}^{\prime }nx_{i,t‐j}+\mu_{i}+\varepsilon_{\mathrm{it}}$$ (2)

Where *y* is the size of the shadow economy (a per cent of GDP) and used as a scalar response variable, *x* is the vector of all the independent variables (i.e. GDP per capita, trade openness, bank credit, government expenditure, and inflation) as expressed in Eq (1). The coefficients of the lagged dependent and independent variables are mentioned by *θ* and *γ*, respectively. Similarly, fixed effect and stochastic error terms are mentioned by *μ* and *ε*, respectively. The subscripts *i*(*i*−1,2,….*N*) and *t*(*t*−1,2,…1) are used to denote the entities and time period, respectively. For computing the error correction model, Eq (2) is to be converted into Eq (3), which is mentioned below:

$$\Delta ny_{it}=\Psi_{i}ECT_{it}+\sum_{j=1}^{p}\theta_{ij}\Delta ny_{i,t-j}+\sum_{j=0}^{q}\gamma_{ij}^{\prime }\Delta nx_{i,t-j}+\mu_{i}+\varepsilon_{it}$$ (3)

Where Ψ_*i*,*t*−1_ is the error correction term $\left(ECT_{it}=ny_{it-1}-\alpha_{i}nx_{it-j}\right)$ or speed with which it adjusts toward the long-run equilibrium where negative coefficient (−*α*_*i*_) is required to establish the stable equilibrium. The PMG model requires homogeneity in the long-run coefficient, i.e. *λ* = *λ*_*i*_ for all the cross-section units, and the same needs to be tested empirically [79]. To calculate the unknown coefficient $\Psi_{i}=-(\sum_{j=1}^{p}\theta_{ij})$ maximum likelihood approach is used. However, the PMG analysis allows for heterogeneity among all the short-run coefficients [79]. The estimators are expressed through Eq (4).


$$\begin{array}{ll} \alpha_{PMG} & =\frac{\sum_{i=1}^{N}\alpha_{i}}{N};\beta_{PMG}=\frac{\sum_{i=1}^{N}\beta_{i}}{N};\theta_{jPMG}=\frac{\sum_{i=1}^{N}\theta_{ij}}{N},\\ \;j & =1,2\ldots ,p-1;\gamma_{jPQG}=\frac{\sum_{i=1}^{N}\gamma_{ij}}{N},j=1,2\ldots ,q-1 \end{array}$$ (4)

Moreover, our study recruits the Granger causality test for panel data to examine causality between independent variables with the shadow economy.

### 3.2 Data

This study uses a sample of 8 ASEAN countries, including Brunei Darussalam, Cambodia, Indonesia, Malaysia, the Philippines, Singapore, Thailand, and Vietnam, from 2000 to 2017. Most of these countries are emerging markets except for a tiny country Brunei and the city-country, Singapore. Table 1 summarises the measurement of the variables used in this paper and relevant data sources.

**Table 1:** Description of variables and measurement.

No.	Variable	Measurement	Abbreviation	Source
*Dependent variable*				
1	Shadow economy	Shadow economy (per cent of GDP)	SE	[5]
*Independent variables*				
2	National intellectual capital	Index of national intellectual capital	INIC	[6, 80]
*Control variables*				
3	Economic growth	Natural logarithm of GDP per capita (constant 2010 US)	LGDPP	[80]
4	Trade openness	Trade (per cent of GDP)	TR	[80]
5	Bank credit	Domestic credit provided by the banking sector (per cent of GDP)	CE	[80]
6	Government expenditure	General government final consumption expenditure (per cent of GDP)	GE	[80]
7	Inflation	The inflation rate, consumer prices	INF	[80]

Table 2 presents the descriptive statistics of all variables. The highest lowest values of the shadow economy are 0.546 and 0.094, or 54.6 per cent and 9.4 per cent of the national GDP during the period of research and across countries in the sample. The average size of the shadow economy of the ASEAN countries is about 0.3024. The mean value of national intellectual capital, proxied by the INIC, is 0.4104 with a standard deviation of 0.1775, a minimum of 0.0634, and a maximum of 0.8947.

**Table 2:** Descriptive statistics.

	Observations	Mean	Min	Max	Std. Dev.
**SE**	144	0.3024	0.0940	0.5460	0.1301
**Δ SE**	136	-0.0032	-0.0890	0.0830	0.0175
**INIC**	144	0.4104	0.0634	0.8947	0.1775
**ΔINIC**	136	0.0165	-0.0280	0.1506	0.0190
**CE**	144	0.6681	0.0598	1.3072	0.3865
**ΔCE**	136	0.0153	-0.1299	0.2044	0.0592
**TR**	144	1.4611	0.3742	4.3732	0.9486
**ΔTR**	136	-0.0073	-0.7913	0.4303	0.1225
**LGDPP**	144	8.5063	6.0600	10.9600	1.4179
**ΔLGDPP**	136	0.0339	-0.0385	0.1179	0.0286
**GE**	144	0.1128	0.0346	0.2939	0.0542
**ΔGE**	136	0.0005	-0.0547	0.0614	0.0122
**INF**	144	0.0353	-0.0231	0.2499	0.0401
**ΔINF**	136	0.0004	-0.2565	0.1732	0.0416

**Notes: SE:** Shadow economy; **INIC**: Index of national intellectual capital; **CE**: Bank credit; **TR**: Trade openness; **LGDPP**: GDP per capita**; GE**: Government expenditure; **INF**: Inflation

*Source*: Authors’ calculations.

**Fig 1:**
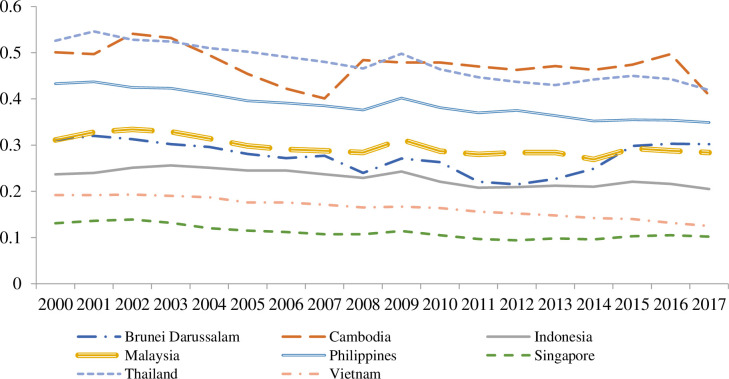
The size of the shadow economy for the ASEAN countries for almost two decades, from 2000 to 2017.

Fig 1 presents the size of the shadow economy of each country across the years. Overall, Singapore has the smallest shadow economy size, while Thailand witnessed a decreasing trend from 2000 to 2017. In addition, the shadow economy in Cambodia has fluctuated through this period and reached the peak of 0.546 (54.6 per cent of the national GDP) in 2016. Vietnam appears to have the smallest size of the shadow economy, which is approximately 20 per cent of the national economy in the region.

Table 3 shows the pairwise Pearson correlations between the variables. We note the negative and significant correlation between national intellectual capital and the shadow economy. This correlation initially indicates that the higher level of the national intellectual capital of nations is associated with the smaller shadow economy.

**Table 3:** The correlation matrix.

	SE	INIC	CE	TR	LGDPP	GE	INF
**SE**	-						
**INIC**	-0.469***	-					
**CE**	0.257**	0.675***	-				
**TR**	-0.4968***	0.645***	0.412**	-			
**LGDPP**	-0.429***	0.7762***	0.391***	0.526***	-		
**GE**	0.0698*	0.348***	0.090	-0.098	0.685***	-	
**INF**	0.0878**	-0.329***	-0.179**	-0.168**	-0.420***	-0.458***	-

Notes

*** significant at 1 per cent level

** significant at 5 per cent level.

**SE:** Shadow economy; **INIC**: Index of national intellectual capital; **CE**: Bank credit; **TR**: Trade openness; **LGDPP**: GDP per capita**; GE**: Government expenditure; **INF**: Inflation.

*Source*: Authors’ calculations.

## 4. Empirical results and discussions

### 4.1 Cross-sectional dependence test

Cross-sectional dependence, which may cause inefficiency in the results, frequently happens in the panel data estimation. The [78] test is used to confirm cross-sectional dependence in this study. Table 4 shows the results of the test. At the 1 per cent significance level, the hypothesis of cross-sectional dependence cannot be accepted. This finding indicates that the panel unit root test is more reliable when the first difference of variables is used in the analysis.

**Table 4:** Cross-section dependence test results.

Variables	SE	INIC	CE	TR	LGDPP	GE	INF
CD test	16.159***	21.333***	9.145***	2.801***	12.192***	5.217***	10.992***
*p*-value	0.000	0.000	0.000	0.000	0.000	0.000	0.000

Notes

*** significant at 1 per cent level.

**SE:** Shadow economy; **INIC**: Index of national intellectual capital; **CE**: Bank credit; **TR**: Trade openness; **LGDPP**: GDP per capita**; GE**: Government expenditure; **INF**: Inflation.

*Source*: Authors’ calculations.

### 4.2 Panel unit root test

The panel unit-root test is employed to examine the stationarity of all variables. We utilize the panel unit-root test [81] suggested to examine the stationarity and determine the concerned variables’ integration order. As presented in Table 5, all variables are stationary at the first difference. These results imply that a long-run co-integrating relationship among the variables used in our analysis is possible.

**Table 5:** Panel unit root test results.

Variables	Level		First Difference		Order of Integration
	Constant (1)	Constant and Trend (2)	Constant (3)	Constant and Trend (4)	
**SE**	0.622 (0.733)	1.596 (0.945)	-1.797** (0.036)	-6.128*** (0.000)	I (1)
**INIC**	-0.561 (0.287)	1.061 (0.856)	-1.675** (0.047)	-2.439*** (0.007)	I (1)
**CE**	0.374 (0.646)	2.012 (0.978)	-3.670*** (0.000)	-3.256*** (0.001)	I (1)
**TR**	1.545 (0.939)	2.345 (0.990)	-1.995** (0.023)	-1.939*** (0.000)	I (1)
**LGDPP**	1.265 (0.897)	2.379 (0.991)	-3.013*** (0.000)	-2.572*** (0.005)	I (1)
**GE**	-0.721 (0,235)	1.390 (0.918)	-2.809*** (0.002)	-2.375*** (0.009)	I (1)
**INF**	-0.038 (0.485)	0.485 (0.686)	-5.474*** (0.000)	-5.098*** (0.000)	I (1)

Notes

*, **, *** significant at 10 per cent, 5 per cent and 1 per cent levels, respectively. The p-values are shown in parentheses. The Z[t-bar] is reported.

**SE:** Shadow economy; **INIC**: Index of national intellectual capital; **CE**: Bank credit; **TR**: Trade openness; **LGDPP**: GDP per capita**; GE**: Government expenditure; **INF**: Inflation.

*Source*: Authors’ calculations.

### 4.3 Panel cointegration test

Results from our unit-root tests confirm that all variables used in our analysis are integrated at I(1). As such, these variables may move together in the long run. In particular, national intellectual capital and shadow economy are co-integrated. Our study uses various panel cointegration tests, including [82–85] residual cointegration tests. Table 6 presents the results from these tests. Our results confirm that the null hypothesis of no cointegration cannot be accepted at the 5 per cent significance level. This finding implies a long-run equilibrium relationship between the shadow economy and national intellectual capital. Moreover, a long-run equilibrium relationship indicates that Granger causality analysis should be considered in this study.

**Table 6:** Results of the cointegration test.

	Statistics
*Pedroni*	
Modified Phillips-Perron t	3.6901***
Phillips-Perron t	-3.0545**
Augmented Dickey-Fuller t	-1.9536**
*Kao*	
Modified Dickey-Fuller t	-11.4959***
Dickey-Fuller t	-8.3687***
Augmented Dickey-Fuller t	-5.4681***
Unadjusted modified Dickey-Fuller t	-12.1111***
Unadjusted Dickey-Fuller t	-8.4256***
*Westerlund*	
Variance Ratio	3.9637***

Notes

**, *** significant at 5 per cent and 1 per cent levels, respectively

*Source*: Authors’ calculations.

### 4.4 Empirical findings on the relationship between shadow economy and national intellectual capital

We employ the panel DOLS estimator suggested by [86] and the panel FMOLS estimator developed by [87] to examine the relationship between shadow economy and national intellectual capital. The results in Table 7 indicate that national intellectual capital impact negatively and significantly on the size of the shadow economy. This finding implies that improving the national intellectual capital will reduce the shadow economy size of the ASEAN countries. Findings from previous studies confirm the positive relationship between national intellectual capital and economic growth [16, 21]. Economic growth creates jobs and reduces poverty [53, 63], leading to a reduction in the shadow economy. Our findings support the dualism and voluntarism schools of thought on informality. This finding demonstrates that the shadow and formal economies are substitutes [65]. In addition, an increase in trade openness appears to reduce the shadow economy for the ASEAN countries. This result is supported by [13, 88]. We consider that trade openness improves productivity and creates more jobs in the official sector, decreasing the shadow economy.

**Table 7:** Empirical findings on the effects of national intellectual capital on the shadow economy using the DOLS and FMOLS estimations.

	DOLS	FMOLS
**INIC**	-0.229***	-0.377***
**CE**	0.155***	0.425***
**TR**	-0.115***	-0.324***
**LGDPP**	-0.202**	-1.267***
**GE**	0.601*	-1.451
**INF**	0.379**	5.875***
**Observations**	128	128
**R-squared**	0.9493	0.9369

Notes

*, **, *** significant at 10 per cent, 5 per cent and 1 per cent levels, respectively.

**INIC:** Index of national intellectual capital; **LGDPP:** GDP per capita; **TR**: Trade openness; **CE:** Bank credit; **GE**: Government expenditure; **INF**: Inflation.

*Source*: Authors’ calculations.

In contrast, an increase in bank credit, government expenditure, and inflation appear to link with the larger shadow economy for the ASEAN countries. First, an increase in the banking sector credit tends to raise the level of liquidity, which will exert more pressure on the money quantity in circulation, resulting in high inflation [13]. Inflation, in turn, brings economic hardships creates incentives to participate in the shadow economy [13, 88]. Second, government spending leads to an increase in the shadow economy because the spending decisions distort the efficient allocation of resources. Third, the increase in government spending in the official economy causes the crowding-out effects and distorts competition in the market [37]. Thus, increasing government spending encourages individuals and firms to move their activities into the shadow economy. Our result aligns with findings from [13, 88].

### 4.5 Robustness check

The pooled mean group (PMG) method examines the effective long-term estimates and consistent mean values when the sample sizes are large [89, 90]. In this paper, we also use the PMG technique to ensure the robustness of the empirical findings. Empirical findings are presented in Table 8. Our results reconfirm the negative and significant effect of national intellectual capital on the size of the shadow economy. An increase in national intellectual capital is associated with a reduction in the shadow economy in the long run in the ASEAN countries. Furthermore, the estimated economic growth and trade openness coefficients are negative and significant. These results mean that the higher the economic prosperity and integration, the lower the shadow economy. However, these effects cannot be observed in the short run, except for trade openness. In addition, an expansion of bank credit and government expenditure will lead to an increase in the shadow economy in the long run. These results reconfirm our results, as presented in Table 6. The same effects can also be observed in the short run for an expansion of bank credit.

**Table 8:** Empirical findings on the effects of national intellectual capital on shadow economy using the pooled mean group estimation.

Variables	Coefficient	Prob*
*Long-run coefficients*		
**INIC**	-0.249	0.000***
**LGDPP**	-0.116	0.000***
**TR**	-0.036	0.000***
**CE**	0.135	0.000***
**GE**	0.911	0.000***
**INF**	-0.089	0.140
**Error correction coefficients**	-0.523	0.065*
*Short-run coefficients*		
**D(INIC)**	0.009	0.890
**D(LGDPP)**	-0.076	0.704
**D(TR)**	-0.058	0.001***
**D(CE)**	0.151	0.037**
**D(GE)**	0.732	0.116
**D(INF)**	0.043	0.073*
**C**	0.653	0.064*
**Observations**	128	

Notes

*, **, *** significance at 10%, 5% and 1% level, respectively.

**INIC:** Index of national intellectual capital; **LGDPP:** GDP per capita; **TR**: Trade openness; **CE:** Bank credit; **GE**: Government expenditure; **INF**: Inflation.

*Source*: Authors’ calculations.

### 4.6 The causality relationship between shadow economy and national intellectual capital using the panel Granger causality test

**Fig 2:**
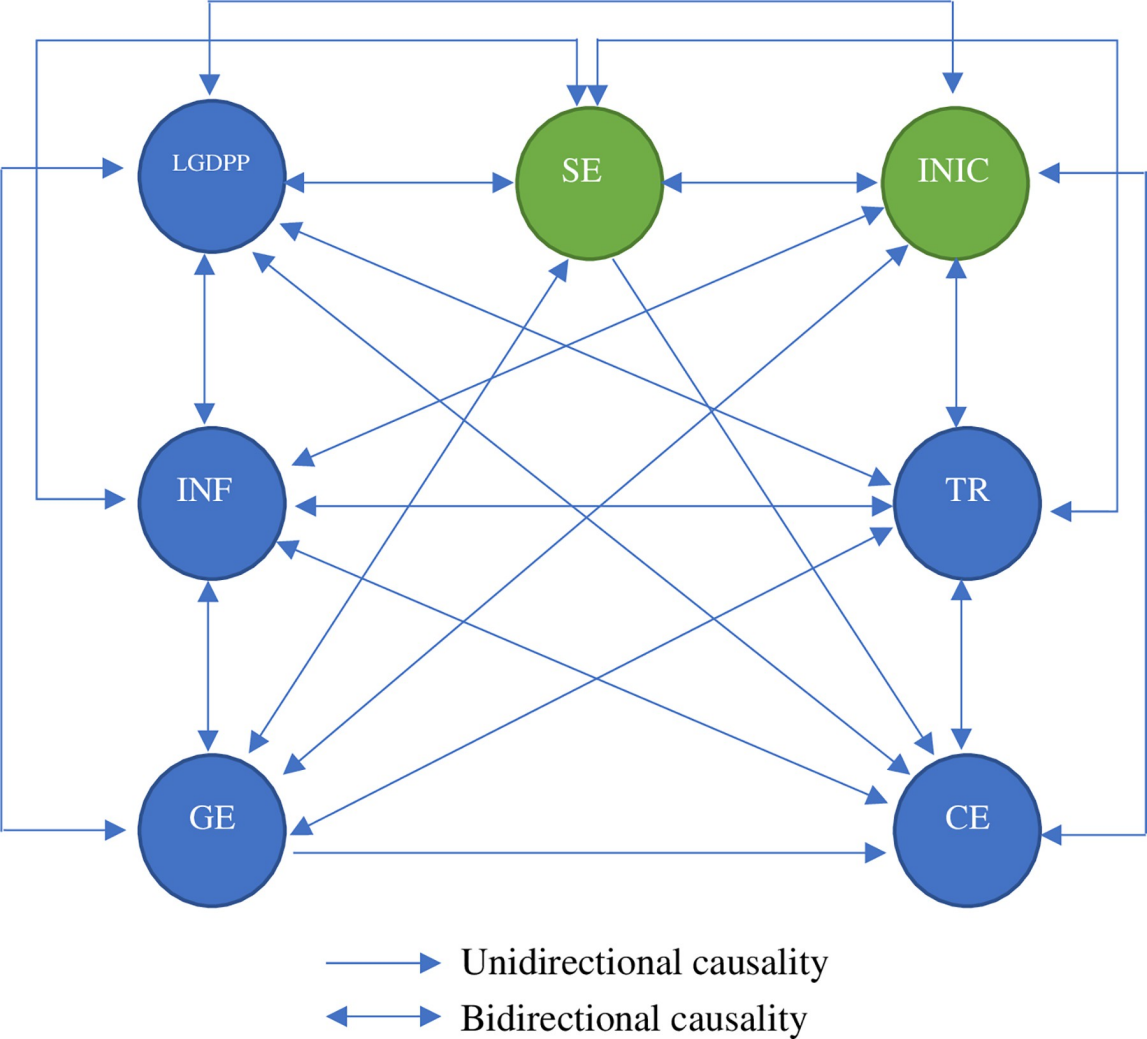
Causality relationship flows. ***Notes*: SE:** Shadow economy; **INIC:** Index of national intellectual capital; **LGDPP:** GDP per capita; **TR**: trade openness; **CE:** Bank credit; **GE**: Government expenditure; **INF**: Inflation.

Our empirical results indicate that an improved national intellectual capital will reduce shadow economy size in the ASEAN countries. We now examine the causality relationship between national intellectual capital and the shadow economy. We employ a panel causality method proposed by [91] to detect the direction of the causality. Table 9 shows that a bi-directional causality relationship between national intellectual capital and shadow economy does exist. Fig 2 illustrates the results of the causality relationship graphically.

**Table 9:** Empirical results on the causality relationship between shadow economy and national intellectual capital.

Hypothesis	Z-bar	Z-bar tilde	Conclusion
INIC → SE	10.5053***	1.7776*	Bidirectional causality between national intellectual capital and the shadow economy
SE → INIC	1.9622**	-0.1598	
LGDPP → SE	8.0579***	5.8071***	Bidirectional causality between economic growth and the shadow economy
SE → LGDPP	16.4647***	3.1291**	
TR → SE	5.1562***	3.6257***	Bidirectional causality between trade openness and the shadow economy
SE → TR	20.0529***	3.9428***	
CE → SE	-0.1574	-0.3690	Unidirectional causality from a shadow economy to bank credit
SE → CE	10.4343***	7.5935**	
GE → SE	3.9745***	2.7373**	Bidirectional causality between government expenditure and the shadow economy
SE → GE	6.1920***	4.4043***	
INF → SE	3.6650***	2.5046**	Bidirectional causality between inflation and shadow economy
SE → INF	3.9927***	2.7510**	
INIC → LGDPP	6.9381***	4.9652***	Bidirectional causality between national intellectual capital and economic growth
LGDPP → INIC	7.9546***	5.7294***	
TR → INIC	3.2001**	2.1551**	Bidirectional causality between national intellectual capital and trade openness.
INIC → TR	1.9893**	1.2449	
CE → INIC	7.5011***	1.0963	Bidirectional causality between national intellectual capital and bank credit
INIC → CE	10.6264***	7.7379***	
GE → INIC	1.8080*	0.3267	Bidirectional causality between national intellectual capital and government expenditure
INIC → GE	4.6162***	0.4421	
INF → INIC	-0.7492	-0.8138	Unidirectional causality from national intellectual capital to inflation
INIC → INF	3.0995**	2.0795**	
TR → LGDPP	4.6704***	1.7578*	Bidirectional causality between trade openness and economic growth
LGDPP → TR	11.7825***	7.2800***	
LGDPP → CE	11.9260***	8.7149**	Bidirectional causality between economic growth and bank credit
CE → LGDPP	2.1406**	0.9869	
GE → LGDPP	19.6800***	3.8583***	Bidirectional causality between government expenditure and economic growth
LGDPP → GE	3.2348***	2.1812**	
INF → LGDPP	17.1775***	3.2907**	Bidirectional causality between economic growth and inflation
LGDPP → INF	3.5014***	2.3817**	
CE → TR	12.4046***	2.2084**	Bidirectional causality between bank credit and trade openness
TR → CE	8.2591***	3.5522***	
GE → TR	16.1322***	3.0537***	Bidirectional causality between government expenditure and trade openness
TR → GE	3.7983***	2.6048***	
INF → TR	5.9439***	2.3946**	Bidirectional causality between inflation and trade openness
TR → INF	6.9108***	4.9447***	
GE → CE	3.3988***	1.8081*	Unidirectional causality from government expenditure to bank credit
CE → GE	-0.3496	-0.5134	
INF → CE	3.2944***	1.7399*	Bidirectional causality between inflation and bank credit
CE → INF	3.6835***	2.5185**	
GE → INF	3.9525***	2.1695**	Bidirectional causality between government expenditure and inflation
INF → GE	12.4261***	7.7001***	

Notes

*, **, *** significant at 10 per cent, 5 per cent and 1 per cent levels, respectively

A → B denotes unidirectional Granger causality running from A to B.

**SE:** shadow economy; **INIC:** Index of national intellectual capital; **LGDPP:** GDP per capita; **TR**: Trade openness; **CE:** Bank credit; **GE**: Government expenditure; **INF**: Inflation.

*Source*: Authors’ calculations.

## 5. Concluding remarks and policy implications

Reducing the shadow economy size is always one of the fundamental macroeconomic policies for governments worldwide, especially for the ASEAN countries, which have exhibited a significant size of the shadow economy. Previous studies have examined the effect of various macroeconomic indicators on the shadow economy. However, the effect of the national intellectual capital on the shadow economy has largely been ignored in the existing literature. This study uses a sample of eight members of the ASEAN over the 2000–2017 period to determine the drivers of the shadow economy and the effect of national intellectual capital on the shadow economy. Our study also examines the short-run and long-run effects and the Granger causality relationship between them.

Our empirical results are two folds. The first group of results focuses on the effect of national intellectual capital on the shadow economy and their Granger causality relationship. Results in this group can be summarised as follows. First, we find that the national intellectual capital and shadow economy are co-integrated in the long run. This finding implies that the national intellectual capital and shadow economy have a relationship in the long run. Second, our empirical findings indicate that a more significant accumulation of national intellectual capital reduces the shadow economy size in the long run. Third, there exists a bi-causality relationship between national intellectual capital and the shadow economy. This finding indicates that policies targeting the shadow economy may also affect the national intellectual capital. The opposite conclusion also holds.

The second group of our empirical findings focuses on the shadow economy drivers. For the Southeast Asian countries, the key drivers leading to the reduction of the shadow economy include trade openness and economic growth. Meanwhile, an increase in bank credits and government expenditures is associated with an increased shadow economy. A bidirectional causality relationship is also found between the shadow economy and critical macroeconomic factors such as economic growth, trade openness, and government expenditure. Our findings indicate that policies supporting economic growth and trade openness will also limit the expansion of the shadow economy.

Policy implications have emerged based on the results of this study. First, the governments of the Southeast Asian countries should formulate and implement policies to improve the accumulation of national intellectual capital by investing in human capital and encouraging the development of intellectual infrastructure. These policies will also play an essential role in reducing the shadow economy size in the long term. Second, policies related to promoting economic cooperation, attracting foreign investment, promoting trade, and increasing trade openness should also be planned. Firms can take advantage of international trade by opening up, thereby reducing the incentive for entrepreneurs to operate in the informal sector. Hence, the size of the shadow economy can be controlled. Third, our empirical results have also shown that economic growth encourages a reduction in the shadow economy size. As such, policymakers should consider promoting economic growth and, as a result, employment for the people. Doing so is expected to reduce the size of the shadow economy because people with good income sources from the official economy will no longer be interested in working in the shadow economy. Fourth, the governments should carefully consider implementing monetary and fiscal policies with a clear understanding that an increase in bank credit and government expenditure may lead to a larger shadow economy.

This study suffers limitations. Limited data is worth mentioning. Future studies may benefit from using historical data for an extended period on the shadow economy. A new theoretical framework may need to be developed to explain the channels through which national intellectual capital impacts the shadow economy. In addition, the effects of national intellectual capital on the shadow economy are currently under-examined. This effect may be relevant and important to be investigated for other countries/ regions in the future.
